# Optimal tillage depths for enhancing rice yield, quality and lodging resistance in the rice production systems of northeast China

**DOI:** 10.7717/peerj.15739

**Published:** 2023-07-19

**Authors:** Diankai Gong, Guijin Dai, Ying Chen, Guangxing Yu

**Affiliations:** Liaoning Rice Research Institute, Shenyang, China

**Keywords:** Breaking resistance, Grain yield, Lodging index, Root system, Ploughing depth

## Abstract

Long-term shallow tillage leads to poor development of root system and deterioration of soil quality. Field experiments were conducted during 2019–2021 to explore the effects of different tillage depths on rice root system, stem lodging resistance, rice yield and quality. The experimental treatments were comprised of four tillage depths *i.e*., 14 cm (TD 14) as the control, 17 cm (TD17), 20 cm (TD20), and 23 cm (TD23) by using a tractor- mounted hydraulic-adjustable. Results indicated that the TD17 treatment substantially improved the breaking resistance by 39.45–72.37% and decreased the lodging index by 11.73–29.94% of first to third node attribute, increased the stem diameter and unit length dry weight and decreased the internode length, compared with control. The TD17 treatment also reduced the chalkiness, chalkiness rate by 26.23% and 32.30%, respectively. Moreover, the viscosity value and cooking and eating quality of rice in TD17 treatment were improved 27.30% and 12.33%, respectively, compared to control. Moreover, the TD20 treatment enhanced the grain yield by 9.18% owing to the higher panicle number and grain number per panicle. The highest photosynthetic rate was also found in the TD20 treatment, which was significant higher 15.57% than TD14 treatment. Overall, the 17–20 cm was found the optimum tillage depth and therefore recommended to the farmers to get improved rice yield with minimum lodging in the rice production systems of the Northeast China.

## Introduction

In agricultural production systems, tillage plays an important role in ensuring the growth and development of crops, transporting water and nutrients, and maintaining nutrient cycling and transformation (Fahad et al., 2022; Elkhlifi et al., 2023; Ishfaq et al., 2020). Different tillage methods have different effects on soil physicochemical properties, which in turn affect crop growth, yield and quality (Khan et al., 2023; He et al., 2020). At present, rotary and surface tillage are commonly used in rice growing areas of Northeast China, which generally operates only in the top 15 cm soil layer. Saini et al. (2022) reported that long-term surface tillage could lead to shallower plough layers, thicker plough bottom layers, hardened soil, decreased porosity, decreased soil storage capacity, and decreased soil fertilizer supply and nutrient retention capacity.

No doubt, tillage depth has a significant impact on soil properties, water and fertilizer reserves and crop growth and yield. For example, deep tillage significantly reduces the bulk density of the plough layer and increases the soil porosity, thus increasing the water storage capacity of the soil (Qiang, Sun & Ning, 2022). Deep tillage in paddy fields can increase the water content of the top layer (0–20 cm) of the soil and improve crop yields (Zhao et al., 2018). However, improper tillage can limit the surface area of rice root system and its ability to withstand against harsh environments which ultimately affects yield and quality of crops. The increase in tillage depth often results in a significant increase in net soil displacement that leads to the thickening and improvement of the tilled soil layer. Good agricultural practices (GAPs) can optimize the water and air permeability, increase the conversion rate of mineral nutrients (Wang et al., 2021), and improve the activity of microorganisms in the soil (Chen, Dikgwatlhe & Xue, 2015). Therefore, GAPs improve the ability of roots to absorb nutrients from the soil and ensure improved rice productivity (Huang, Zou & Jiang, 2012; Liu, Ping & Li, 2010; Chen et al., 2021a).

Furthermore, the demand for rice with excellent eating quality in China has increased sharply (Li et al., 2021a; Ashraf et al., 2022), however, good quality rice cultivars are generally poor in lodging resistance (Li et al., 2021b). In addition to improving the lodging resistance through cultivar development, it is imperative to adopt reasonable tillage measures to improve the lodging resistance in rice (Gong et al., 2011; Liu et al., 2020).

Current research on different tillage depths has mainly focused on soil physicochemical properties and soil nutrients, whereas a little is known about the effects of different tillage depth on grain quality and lodging resistance, therefore a comprehensive research is required explore the inter-relationships of rice root system, stem lodging resistance, yield and quality of rice under different tillage depths. Therefore, the present study is conducted to assess the effects of different tillage depths on the yield, quality and lodging resistance in rice to provide a theoretical basis and practical applications for the rational farming measures in rice production areas of Northeast China.

## Materials and Methods

### Experimental details

The field experiments were conducted in 2019–2021, at Liaoning Rice Research Institute, Shengyyang City, Liaoning Province, China. The soil physicochemical properties of the experimental site are shown in Table 1. The experimental treatments were comprised of four tillage depths *i.e*., 14 cm (TD14) as the control, 17 cm (TD17), 20 cm (TD20), and 23 cm (TD23). The 2-year field experiments were conducted in completely randomized complete block design with three replications. A tractor-mounted hydraulic-adjustable tiller was adjusted to the required depths for tillage operations. The seeds of locally grown high-yielding rice cultivar ‘Liaojing 212’ was sown for nursery raising on April 22, and transplanted on May 24 at 30 × 16.5 cm spacing during both years. The net plot size of each experimental unit was 1,000 m^2^. The urea, diammonium phosphate (DAP), and potassium sulfate was applied at 225, 150 and 150 kg ha^−1^. The urea was applied in three splits with a ratio of 5:3:2 as base:tiller:ear fertilizer = 5:3:2. The DAP was applied once as base fertilizer whereas potassium sulfate was applied with a ratio of 1:1 as base:tiller fertilizer. The alternate wetting and drying was practiced for irrigation purpose whereas guidelines of the local government were followed for pest management.

**Table 1:** Basic physical and chemical properties of soil.

Soil location	Total nitrogen (g/kg)	Total phosphorus (g/kg)	Total potassium (g/kg)	Alkalined nitrogen (mg/kg)	Available phosphorus (mg/kg)	Available potassium (mg/kg)	Organic matter (g/kg)
Tillage layer	14.39	0.78	21.92	157.02	25.77	229.80	20.59
Plow bottom	7.71	0.65	20.16	77.53	16.42	145.84	14.85

### Observations

#### Morphological indices of rice root system

The root sampling measurements were accomplished according to Pan et al. (2016) with some modifications. Each planting plot requires the filling of three net bags (25 cm × 30 cm) with slurry distributed equally. Afterward, the rice seedlings are transplanted into each net bag. Transplanting must be done with care to prevent any harm to the roots and guarantee that the water and nutrients are efficiently absorbed by the rice roots following transplantation. At full heading stage, three rice hills were selected and washed. The root analysis instrument WinRhizo-LA1600 (Regeng Instuments Inc., Quebec, Canada) was used to analyze the root length, root diameter, root surface area and root volume and other root morphological traits.

#### Determination of the lodging index

The stem lodging and its physical characteristics were determined according to Pan et al. (2019). In brief, at 30 days after the full heading stage, three hills were selected from each treatment to measure the plant height, height of center of gravity, panicle length, panicle weight and panicle stalk angle of rice. Subsequently, the bending-resistant strength of each internode was measured using a stalk strength tester (Model: YYD-1). The stem internode distance, panicle length, dry varieties, the bending resistant strength between the first nodes (N1), second nodes (N2), third nodes (N3) below the neck of the panicle, and the length and fresh weight from the base of each internode to the tip of the panicle were measured. The bending moment and lodging index between N1, N2, and N3 in each treatment were calculated as follows:

Bending moment = length from internode base to panicle tip (cm) × fresh weight of the part from internode base to panicle tip (g)

Lodging index = bending moment/bending resistant force × 100

#### Photosynthetic characteristics

At 30 days after the full heading stage, the photosynthetic rate of flag leaves was measured using a portable photosynthesis instrument (LI-6400; LI-COR Biosciences, Lincoln, NE USA), Five-point sampling method was adopted in each plot, and five representative flag leaves were selected from each point, and each flag leaf was measured three times to obtain the average value.

#### Yield and its components

The rice yield was determined according to Zhou et al. (2019). In brief, the yield was measured at an area of 5 m^2^ per treatment. After drying and threshing, the rice grains were weighed and converted into the actual yield. Meanwhile, the number of effective panicles per 20 consecutive hills in each plot was noted, and five hills of rice were collected to estimate the number of grains per panicle, seed setting rate and 1,000-grain weight.

#### Rice quality characters

The milling quality were determined according to Bao et al. (2018). The appearance quality was determined by WanShen SCE rice appearance quality system, the eating quality was measured by Japanese Satake rice taste meter, the content of amylose in rice was determined by sulfuric acid anthracene copper method. The protein content in rice was measured by FOSS Kjeldahl analyzer. The VA (Rapid Visco Analyzer) was used to test the viscosity of rice flour starch in the samples in accordance with the standards (American Association of Cereal Chemist, 1999) regulated by American Association of Cereal Chemists, with the use of Thermal Cycle Windows (TCW) software.

### Experimental design and data analysis

The 2-year field experiments were conducted in randomized complete block design with three replications. The measured data were processed and analyzed by Microsoft Excel 2007 and SPSS18. One-way analysis of variance was used whereas Duncan’s new multiple range test was used to separate treatment means. The data of both years were consistent with no significant difference; therefore, the data were pooled for the statistical analysis.

## Results

### Effects of different tillage depths on the root system of rice

The root system of rice at full heading stage were significantly affected by different tillage depths (Table 2). For example, the total root length of the TD17 treatment was significantly higher than TD20 and TD14, and the TD17 is 37.07% longer than the TD14 treatment. Moreover, the projected area and surface area of the TD17 treatment are the largest, significantly higher than other tillage treatments. The average root diameter increased with tillage depth, and the TD14 treatment was significantly thicker by 12.19%, 21.05%, and 35.29% than TD17, TD20, and TD23, respectively. The root tip number of TD23 was significantly higher than that of TD14 and TD20, however, no significant difference was found between TD23 and TD17 regarding root tip number.

**Table 2:** Effects of different tillage depths on root morphology traits of rice.

Treatment	Root length (m)	Projected area (m^2^)	Surface area (m^2^)	Mean diameter (mm)	Root volume (cm^3^)	Root tip number
TD14	154.18c	0.07b	0.21b	0.46a	24.24ab	69,590b
TD17	211.33a	0.08a	0.27a	0.41b	27.46a	111,036a
TD20	178.66b	0.07b	0.21b	0.38b	20.87bc	87,496b
TD23	196.53ab	0.07b	0.21b	0.34c	17.15c	112,971a
Y	ns	ns	ns	ns	ns	ns
TD	*	ns	ns	ns	*	*
Y*TD	ns	ns	ns	ns	ns	ns

**Note:**

Different letters in the same row within the same means the significant differences in *P* < 0.05. * indicate significant correlations at the 0.05 levels. TD, tillage depth; TD14, tillage depth 14 cm; TD17, tillage depth 17 cm; TD20, tillage depth 20 cm; TD 23, tillage depth 23 cm; Y, year.

### Effects of different tillage depths on rice stems and lodging resistance

The rice stems and lodging resistance were variably affected under different tillage depths (Table 3). As the tillage depth increases, the plant height and the height of the center of gravity were increased at first and then decreased. The fresh weight of single stem was significantly lower in TD23 than in TD20. The difference in the length of the first and third internodes in the TD17, TD20 and TD23 was not significant, but all were significantly lower than those of TD14. The shortest length of N2 were found in TD17 treatment that was significantly lower than the other treatments. With the increase of tillage depth, the stem diameter and unit length dry weight increased first and then decreased and remained significantly higher than TD14.

**Table 3:** Effects of different tillage depths on rice stems.

Treatment	Plant height (cm)	Center of height (cm)	Fresh weight of single stem (g)	N1			N2			N3		
				INL (cm)	SD (mm)	ULDW (g/cm)	INL (cm)	SD (mm)	ULDW (g/cm)	INL (cm)	SD (mm)	ULDW (g/cm)
TD14	104.61a	57.53ab	15.11ab	3.53a	5.32b	0.024b	10.62a	4.71c	0.019b	15.07a	4.31b	0.015b
TD17	104.79a	58.83a	15.38ab	3.08b	5.75a	0.034a	9.63c	5.33a	0.027a	14.63b	4.80a	0.020a
TD20	103.57ab	57.16ab	16.14a	3.02b	5.91a	0.036a	9.99bc	5.16ab	0.025ab	14.45b	4.61ab	0.019a
TD23	101.43b	56.01b	14.79b	2.86b	5.64a	0.031ab	10.39ab	4.91bc	0.022ab	14.32b	4.45ab	0.016b
Y	ns	ns	ns	ns	ns	ns	ns	ns	ns	ns	ns	ns
TD	*	ns	ns	*	ns	ns	ns	*	ns	*	*	ns
Y*TD	ns	ns	ns	ns	ns	ns	ns	ns	ns	ns	ns	ns

**Note:**

Different letters in the same row within the same rice means the significant differences in *P* < 0.05. * indicate significant correlations at the 0.05 levels. TD, tillage depth; TD14, tillage depth 14 cm; TD17, tillage depth 17 cm; TD20, tillage depth 20 cm; TD 23, tillage depth 23 cm; Y, year. N1, first node; N2, second node; N3, third node; INL, internode length; SD, stem diameter, ULDW, unit length dry weight.

The breaking resistance, bending moment, and lodging index in N1–N3 were significantly affected by different tillage depths (Table 4). It can be noticed that under TD17, the largest breaking resistance in the N1, N2, and N3, the lowest lodging index, and the strongest lodging resistance, the difference with TD14 and TD23 was significant in the N2 and N3. In the N1, the bending moment of TD20 was the largest, which was significantly higher than TD23 treatment. In the N2 and N3, the breaking resistance of TD17 was the largest, which was significantly higher than TD14. Besides, the lodging index of TD14 was the largest in N1to N3, which was significantly higher than that of TD17, TD20, and TD23. On the other hand, no significant differences were found in the N2 and N3.

**Table 4:** Effects of different tillage depths on rice lodging resistance.

Treatment	N1			N2			N3		
	Breaking resistance (N)	Bending moment	Lodging index	Breaking resistance (N)	Bending moment	Lodging index	Breaking resistance (N)	Bending moment	Lodging index
TD14	38.71b	1,518ab	40.39a	16.25b	1,291ab	79.72a	13.13b	994ab	77.35a
TD17	55.32a	1,581a	29.37b	28.01a	1,417a	55.85a	18.31a	1,151a	68.28a
TD20	54.03a	1,609a	31.8b	21.22ab	1,416a	72.16a	15.65ab	1,126ab	72.23a
TD23	45.31ab	1,330b	30.52b	16.49b	1,161b	74.83a	11.25b	915b	87.66a
Y	ns	ns	ns	ns	ns	ns	ns	ns	ns
TD	*	ns	ns	ns	*	ns	*	*	ns
Y*TD	ns	ns	ns	ns	ns	ns	ns	ns	ns

**Note:**

Different letters in the same row within the same rice means the significant differences in *P* < 0.05. * indicate significant correlations at the 0.05 levels. TD, tillage depth; TD14, tillage depth 14 cm; TD17, tillage depth 17 cm; TD20, tillage depth 20 cm; TD 23, tillage depth 23 cm; Y, year. N1, first node; N2, second node; N3, third node.

The relationship among lodging index, breaking resistance and plant height, center of gravity height, fresh weight of single stem, morphological index of root system was shown in Table 5. The N1 and N2 length, root mean diameter was significantly and positively correlated with lodging index of N1 and N2, while the N2 stem diameter had a significant negative correlation with the lodging index of N1 and N2. The N1, N2, and N3 stem diameter was significantly and positively associated with breaking resistance of N1. The fresh weight of single stem, N2 stem diameter, N3 stem diameter, projected root area, and root surface area was significantly and positively correlated with breaking resistance of N3.

**Table 5:** Correlation analysis of buckling resistance, lodging index and plant type characteristics and root morphology.

Parameters	N1		N2		N3	
	Lodging index	Breaking resistance	Lodging index	Breaking resistance	Lodging index	Breaking resistance
Plant height	0.174	0.348	0.088	0.47	0.143	0.459
Center of gravity height	0.349	0.162	0.094	0.291	0.072	0.646*
Fresh weight of single stem	0.198	0.472	0.195	0.253	0.401	0.609*
First node length	0.769**	−0.571	0.695*	−0.552	0.505	−0.509
First node stem diameter	−0.677*	0.697*	−0.507	0.519	−0.166	0.503
Second node length	0.624*	−0.829**	0.607*	−0.753**	0.228	−0.810**
Second node stem diameter	−0.607*	0.817**	−0.621*	0.771**	−0.268	0.820**
Third node length	0.334	−0.369	0.187	−0.294	−0.064	−0.408
Third node stem diameter	−0.259	0.602*	−0.242	0.503	0.013	0.765**
Root length	−0.496	0.442	−0.385	0.439	−0.062	0.415
Projected area	0.048	0.18	−0.234	0.438	−0.285	0.669*
Surface area	0.048	0.18	−0.234	0.438	−0.285	0.669*
Mean diameter	0.694*	−0.383	0.275	−0.106	0.141	−0.139
Root volume	0.402	−0.073	−0.029	0.253	−0.309	0.562
Root tip number	−0.572	0.417	−0.338	0.334	0.098	0.157

**Note:**

* and ** indicate significant correlations at the 0.05 and 0.01 levels. N1, first node; N2, second node; N3, third node.

### Effects of different tillage depths and photosynthetic characteristics and yield

The highest photosynthetic rate (Pn) was found in the TD20 treatment, which was 18.45% higher than TD14 treatment. Moreover, the Pn were decreased with the following trend: TD20 > TD23 > TD17 > TD14. No significant differences were noted in stomatal conductance and intercellular CO_2_ concentration among treatments. Moreover, the transpiration rate of TD14 was significantly higher than TD20 and TD23 treatment (Table 6).

**Table 6:** Effects of different tillage depths on photosynthetic characteristics of rice at grain filling stage.

Treatment	Pn (μmolCO_2_·m^−2^s^−1^)	Cond (μmol H_2_O·m^−2^s^−1^)	Ci (μmolCO_2_·m^−2^s^−1)^	Trmmol (mmol.m^−2^s^−1^)
TD14	13.39b	0.64a	315.93a	5.28a
TD17	15.01a	0.62a	310.25ab	5.32a
TD20	15.86a	0.61a	306.43b	5.36a
TD23	15.45a	0.57a	304.28b	5.28a
Y	ns	ns	ns	ns
TD	*	ns	**	ns
Y*TD	ns	ns	ns	ns

**Note:**

Different letters in the same row within the same rice means the significant differences in *P* < 0.05. * and ** indicate significant correlations at the 0.05 and 0.01 levels. TD, tillage depth; TD14, tillage depth 14 cm; TD17, tillage depth 17 cm; TD20, tillage depth 20 cm; TD 23, tillage depth 23 cm; ; Y, year; Pn, photosynthetic rate; Cond, stomatal conductance; Ci, transpiration rate; Trmmol, intercellular carbon dioxide concentration.

The yield and its related traits were significantly affected by different tillage depths. For example, the highest average yield during both years was remained 10.70 t/ha under the TD20 treatment, which was 9.18% higher than the TD14 treatment. The number of effective panicles per hills were remained as: TD20 > TD17 > TD23 > TD14. No significant difference was noted in 1,000-grain weight, seed setting rate, and grain number per panicle among all treatment (Table 7).

**Table 7:** Effects of different tillage depths on yield and its related traits.

Treatment	Panicle number (panicle/hole)	Grain number per panicle (grains/panicle)	Seed setting rate (%)	1,000-grain weight (g)	Yield (t/ha)
TD14	14.8b	150.1b	89.9a	22.2a	9.8b
TD17	16.1a	157.9a	90.4a	22.2a	10.5ab
TD20	16.9a	156.3a	90.3a	22.3a	10.7a
TD23	15.3ab	154.7a	90.1a	22.2a	10.4ab
Y	ns	ns	ns	ns	ns
TD	*	*	ns	ns	*
Y*TD	ns	ns	ns	ns	ns

**Note:**

Different letters in the same row within the same rice means the significant differences in *P* < 0.05. * indicate significant correlations at the 0.05 levels. TD, tillage depth; TD14, tillage depth 14 cm; TD17, tillage depth 17 cm; TD20, tillage depth 20 cm; TD 23, tillage depth 23 cm; Y, year.

### Effects of different tillage depths on rice quality

The head rice rate, protein, chalky rice rate, and chalkiness degree were affected by different tillage depths. The head rice rate was the highest in TD20 and TD23, which was significantly higher than that in TD14. The same trend was also observed in the chalky rice rate and chalkiness degree with the following trend: TD17 < TD20 < TD23 < TD14. The coarse rice rate, polished rice rate and grain length-width ratio were the largest in the TD17, nevertheless all treatments remained statistically similar (Table 8).

**Table 8:** Effects of different tillage depths on rice processing quality and appearance quality.

Treatment	Coarse rice rate (%)	Polished rice rate (%)	Head rice rate (%)	Chalky rice rate (%)	Chalkiness degree (%)	Length-width ratio
TD14	79.01a	66.54a	59.47b	10.33a	3.22a	1.95a
TD17	80.43a	67.76a	61.52ab	7.62b	2.18b	1.97a
TD20	80.21a	67.68a	61.99a	8.58ab	2.55ab	1.93a
TD23	80.12a	67.61a	62.20a	10.32a	3.08a	1.94a
Y	ns	ns	ns	ns	ns	ns
TD	ns	ns	*	*	*	ns
Y*TD	ns	ns	ns	*	*	ns

**Note:**

Different letters in the same row within the same rice means the significant differences in *P* < 0.05. * indicate significant correlations at the 0.05 levels. TD, tillage depth; TD14, tillage depth 14 cm; TD17, tillage depth 17 cm; TD20, tillage depth 20 cm; TD 23, tillage depth 23 cm; Y, year.

Moreover, the TD17 has the best taste quality, which is 12% higher than the TD14. Moreover, the viscosity and balance of TD17 increased by 27% and 32%, respectively compared with TD14. The TD17 had the highest viscosity and disintegration value, which was higher than that of TD14 by 1.45% and 8.49%, respectively (Table 9). However, the highest setback value and peak time were found in TD14 treatment, which was higher 5.29–57.42% and 11.11–12.71%, respectively, compared to other treatments. However, no significant difference was found in the gelatinization temperature among all treatments (Table 10).

**Table 9:** Effects of different tillage depths on the taste quality of rice.

Treatment	Appearance	Hardness	Viscosity	Balance	Eating value
TD14	3.05a	8.00a	2.93ab	2.90b	46.85b
TD17	3.15a	7.53b	3.73a	3.83a	52.63a
TD20	2.93a	8.03a	2.80b	2.83b	46.23b
TD23	2.63b	8.20a	2.50b	2.45b	44.13b
Y	ns	ns	ns	ns	ns
TD	ns	ns	ns	ns	*
Y*TD	ns	ns	ns	ns	ns

**Note:**

Different letters in the same row within the same rice means the significant differences in *P* < 0.05. * indicate significant correlations at the 0.05 and 0.01 levels. TD, tillage depth; TD14, tillage depth 14 cm; TD17, tillage depth 17 cm; TD20, tillage depth 20 cm; TD 23, tillage depth 23 cm; Y, year.

**Table 10:** Effects of different tillage depths on rice starch viscosity (RVA).

Treatment	Highest viscosity (cP)	Hot pulp viscosity (cP)	Disintegration value (cP)	Cold glue viscosity (cP)	Setback value (cP)	Peak time (min)	Gelatinization temperature (°C)
TD14	2,686ab	1,777a	909b	2,845a	159a	6.37a	72.46a
TD17	2,725a	1,737ab	988a	2,840a	115b	6.30ab	71.92a
TD20	2,694ab	1,727ab	967a	2,795ab	101b	6.31ab	72.03a
TD23	2,609b	1,665b	944ab	2,760b	151a	6.29b	72.45a
Y	ns	ns	ns	ns	ns	*	ns
TD	ns	ns	ns	ns	ns	**	ns
Y*TD	ns	ns	ns	*	*	ns	ns

**Note:**

Different letters in the same row within the same rice means the significant differences in *P* < 0.05. * and ** indicate significant correlations at the 0.05 and 0.01 levels. TD, tillage depth; TD14, tillage depth 14 cm; TD17, tillage depth 17 cm; TD20, tillage depth 20 cm; TD 23, tillage depth; Y, year.

**Figure 1:**
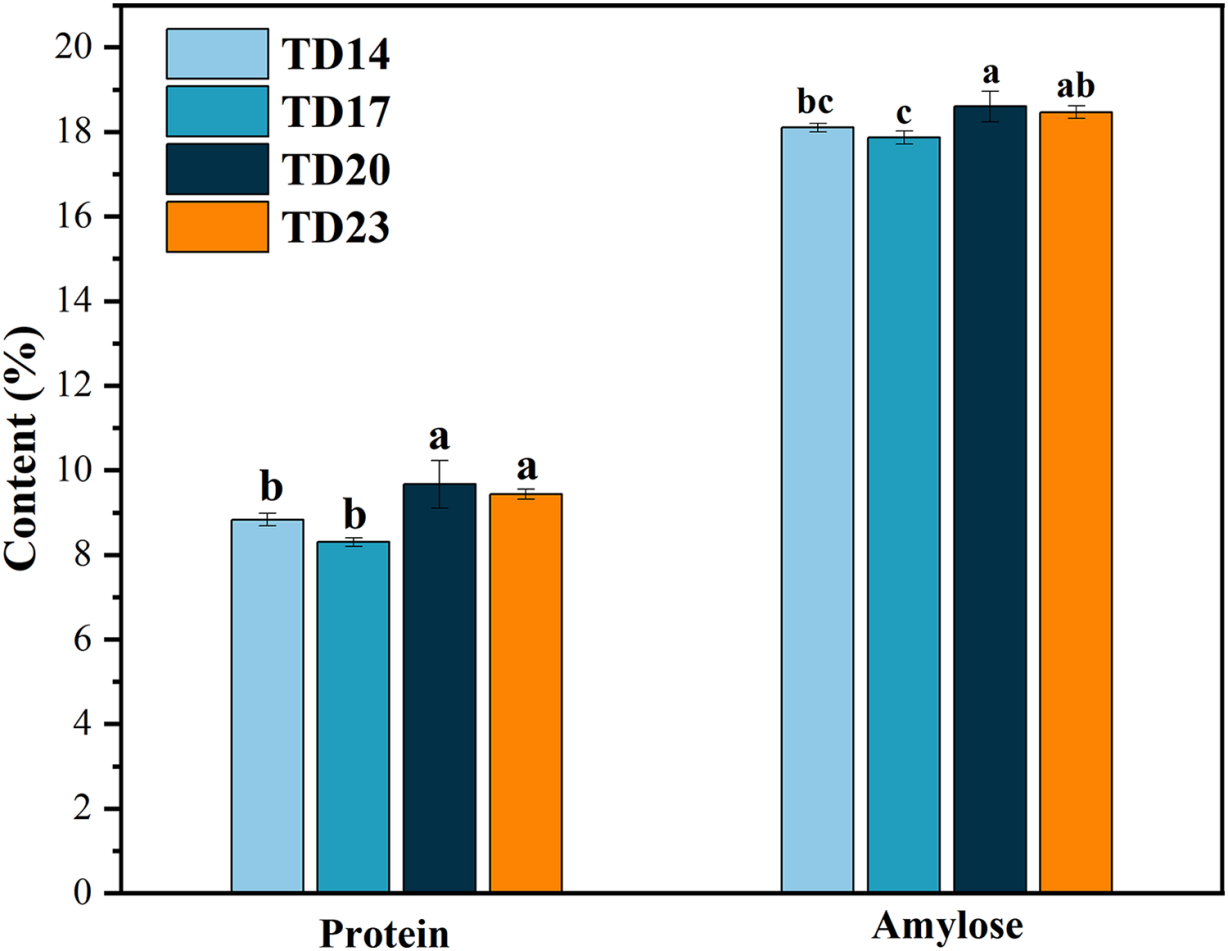
Effects of different tillage depths on the composition of rice. Note: different letters in the same row within the same rice means the significant differences in *P* < 0.05. TD14, tillage depth 14 cm; TD17, tillage depth 17 cm; TD20, tillage depth 20 cm; TD 23, tillage depth.

In addition, significant differences were noted in the amylose and protein contents under different tillage depths. The highest amylose and protein contents were noticed for the TD20 treatment, which was significantly higher than the TD14 treatment. Similar trend in amylose and protein contents was observed *i.e*., TD20 > TD23 > TD14 > TD17 (Fig. 1).

## Discussion

### The effect of tillage on the rice roots and lodging resistance of stems

Different tillage practices play an important role in improving the soil structure, capacity, aeration and permeability, as well as the growth and development of rice (Yao, Teng & Zhang, 2015). The present study revealed the effects of different tillage depths on yield, grain quality, and some lodging resistance in rice. Tillage depth with 17–20 cm greatly improved the breaking resistance and lodging index, thus improved the lodging resistance of rice (Table 4). The morphological structure and mechanical strength of stems play important roles in plant lodging resistance. The present study found that the increments in lodging resistance could be explained by the improvements in the stem diameter and unit length dry weight of N1–N3. Our results agreed with the study of Zhang et al. (2017) who observed that deep tillage reduces the height of the center of gravity of the stem and increases the fresh weight of the stem and thickness of the stem wall of N2. Previously, Dai et al. (2019) found that the deep ploughing can break the bottom of plough, deepen the ploughing layer, improve the soil environment for rice growth, and promote the growth and distribution of the roots. The strong root system can promote the absorption and utilization of nutrients, which is conducive to the increase of stem dry weight and fullness, and the thickening of stem wall at the stem base internode, thus reducing the lodging risk (Li et al., 2023). Furthermore, in present study, it was noticed that most of the physical stem parameters had positive correlations with the breaking-resistance of the internodes and negative correlations with the lodging index (Table 5). These results elucidated that the improvements in the internode diameter, fresh weight of single stem, and unit length dry weight possibly the main factors reducing plant lodging under deep tillage that are in line with the reports by Chen et al. (2021b).

Furthermore, the increase in root morphological traits is also the main reason for the increase in lodging resistance. The present study found that the deep tillage could significantly increase the root length, projected area, and surface area, compared to the control (TD14). The results agreed with Dai et al. (2019), who found that deep tillage can increase the root length density, root surface area density, root tip number and root volume of maize, indicating that deep ploughing was beneficial to the root system to proliferate deep into the soil, compared with rotary tillage. Besides, the root growth and vertical length of the rice root system were significantly increased by deep tillage, and the root activity of rice at the end of tillering increased with the increase of tillage depth (Wang et al., 2017). The root morphological indices *i.e*., the projected area and surface area of the root system was significantly and positively correlated with breaking resistance. The results agreed with Wang et al. (2015), who also found that the lodging index was significantly and negatively correlated with root dry weight, root thickness and number of lateral roots. Under the two cultivation methods of conventional transplanting and throwing seedlings, compared with no-tillage, rice ploughing stalks are more resistant to lodging (Yang et al., 2013). After deep tillage, the roots are easy to grow deep into the soil, with better above-ground growth, stem thickness, increased tillers, and panicle formation. The present study showed that with the increase in ploughing depth, both the plant height and the root average diameter decreased with the ploughing depth. Moreover, the total root length of rice increased at first and then decreased, which possibly due to the deep ploughing to improve the soil structure and bulk density, which is beneficial to the vertical growth of rice roots. When the tillage depth is too deep, the bottom layer of plough is completely broken, which may cause soil nutrient loss and more soil below the plough to turn to the surface, which is not conducive for plant growth. Therefore, a tillage depth of 17–20 cm can improve the growth of below and above-ground plant parts and this improves the lodging resistance of rice.

### Effects of tillage on rice yield and quality

Deep ploughing is generally better regarding rice productivity than shallow rotary tillage and no-tillage (Gu et al., 2017). In present study, the increments in yield could be explained by the improvements in the panicle number and grain number per panicle. The increments in grain yield, panicle number and grain number per panicle might be attributed to the higher net photosynthetic rate and stronger root systems during the heading stage as the rate of net photosynthesis has a decisive role in grain formation mechanism (Sun et al., 2011). The results agreed with Liu et al. (2013), who found that deep tillage (20 cm) can increase the number of grains per ear and seed setting rate of rice, and significantly improve rice yield, compared with normal rotary tillage and shallow tillage. Likewise, Wang et al. (2017) also found that the photosynthetic rate of flag leaves under 20 cm tillage depth was significantly higher than 14 cm tillage depth, which improved the dry biomass, number of effective panicles and grains per panicle, and thus increased rice yield. Deep tillage also improved soil structure, increased root activity, promoted the absorption of nutrients and water by roots, prolonged the photosynthetic period of leaves, and ultimately improved the rice yield. Additionally, deep tillage increases the number of aerobic microorganisms actinomycetes and fungi in the soil. Deep tillage promotes the root, delay the senescence of leaves, prolonged the functional period of the leaves, increases leaf area and plant dry matter, thereby increasing crop yield (Duan, Zhao & Guo, 2007; Lu et al., 2014). Deep tillage is more conducive to increasing the soluble sugar content in leaves than rotary tillage and no-tillage. Therefore, tillage can facilitate early vegetative growth of crops and promote physiological metabolism of plants (Mo et al., 2008). Moreover, the soluble sugar is the carbon source of crop photosynthesis and grain filling (Deng et al., 2023). However, when the deep tillage with more than 20 cm depth, the yield decreased to a certain extent that possibly due to excessive subsoil turned over to the surface, resulting in a decrease in the nutrient content that can otherwise be used by the crop directly. Thus, there was a certain loss of nutrients that led to the reduction in rice yield.

There were few studies on the effects of different tillage practices on rice quality. In this study, it was found that ploughing at a suitable depth was beneficial to reduce the chalkiness and chalkiness rate of rice. Moreover, the TD17 had the lowest amylose and protein content, the highest viscosity, the smallest disintegration value, and the best cooking and eating quality. Deep tillage can increase the content of soil organic matter, increase the number of aerobic microorganisms in the soil, and thus could increase the content of available N in the soil (Liu et al., 2022). Previously, Wang et al. (2016) found that the N content of the aerial parts of plants at the tillering, heading and maturity stage was substantially higher in deep ploughing treatment than that of shallow ploughing. The higher root related parameters were beneficial to lower chalkiness and chalkiness rate, which was in agreement with Li et al. (2021b), who reported that the morpho-physiological traits of rice roots play important roles in grain quality attributes. Deep tillage improves the N supply at the later stages of plant growth and development. Increasing N supply after heading stage decreased rice viscosity and taste quality. However, continuing to increase the tillage depth had a certain negative effect on the cooking quality of rice. In general, viscosity and balance contribute to the taste quality of rice which is directly related to the amylose and protein contents.

## Conclusions

Overall, compared to the TD14, the TD20 enhanced the net photosynthetic rate, and increased the panicle number and grain number per panicle and increased the grain yield of rice. The TD17 reduced the chalkiness, chalkiness rate rice, and improved viscosity value and cooking and eating quality of rice as well as improved the breaking resistance decreased the lodging index first to third node attributed to increase in stem diameter and unit length dry weight and decrease in the internode length, as compared with TD14. Larger root morphological traits such as root length, projected area, surface area, root volume was also observed for TD17 treatment. Therefore, it is recommended to keep the tillage depth between 17–20 cm in order to get higher rice yield with better grain quality and lodging resistance.

## Supplemental Information

10.7717/peerj.15739/supp-1 Supplemental Information 1 Raw data.

## Additional Information and Declarations

### Data Availability

The following information was supplied regarding data availability: The raw data are available in the Supplemental File.
